# Identification of shared fatty acid metabolism related signatures in dilated cardiomyopathy and myocardial infarction

**DOI:** 10.2144/fsoa-2023-0008

**Published:** 2023-03-28

**Authors:** Baoyi Huang, Hanying Lin, Qingfang Zhang, Yujun Luo, Biyao Zhou, Zewei Zhuo, Weihong Sha, Jiahui Wei, Lihua Luo, Huajuan Zhang, Kequan Chen

**Affiliations:** 1 Guangzhou Medical University, Guangzhou, 511436, China; 2 Department of Endocrinology, The First People’s Hospital of Zhaoqing, Zhaoqing, 526020, China; 3 Department of Gastroenterology, Guangdong Provincial People's Hospital (Guangdong Academy of Medical Sciences), Southern Medical University, Guangzhou, 510080, China; 4 Department of Gastroenterology, The Third Affiliated Hospital of Sun Yat-Sen University, Guangzhou, 510630, China; 5 Department of Periodontics, Stomatological Hospital, Southern Medical University, Guangzhou, 510280, China; 6 Department of Gastroenterology, The First Affiliated Hospital of Guangzhou Medical University, Guangzhou, 510120, China

**Keywords:** dilated cardiomyopathy, fatty acid metabolism, immune infiltration, myocardial infarction, nomogram, risk prediction

## Abstract

**Aim:**

It is to be elucidated the risk-predictive role of differentially expressed fatty acid metabolism related genes (DE-FRGs) in dilated cardiomyopathy (DCM) and myocardial infarction.

**Materials & methods:**

Four gene enrichment analyses defined DE-FRGs’ biological functions and pathways. Three strategies were applied to identify risk biomarkers and construct a nomogram. The 4-DE-FRG correlation with immune cell infiltration, drugs, and ceRNA was explored.

**Results:**

DE-FRGs were enriched in lipid metabolism. A risk nomogram was established by *ACSL1, ALDH2, CYP27A1* and *PPARA*, demonstrating a good ability for DCM and myocardial infarction prediction. *PPARA* was positively correlated with adaptive immunocytes. Thirty-five drugs are candidate therapeutic targets.

**Conclusion:**

A nomogram and new biological targets for early diagnosis and treatment of DCM and myocardial infarction were provided.

## Plain language summary

Dilated cardiomyopathy (DCM) and myocardial infarction (MI) are two common types of heart disease. This study investigated the fatty acid metabolism related genes to predict the risk of DCM or MI. Four of them (*ACSL1, ALDH2, CYP27A1* and *PPARA*) were effective in predicting disease risk. Additionally, *PPARA* was found to be associated with immune cell infiltration, and 35 drugs emerged as potential therapeutic targets for these diseases. This study may provide insights into the early diagnosis and treatment of DCM and MI.

## Graphical abstract

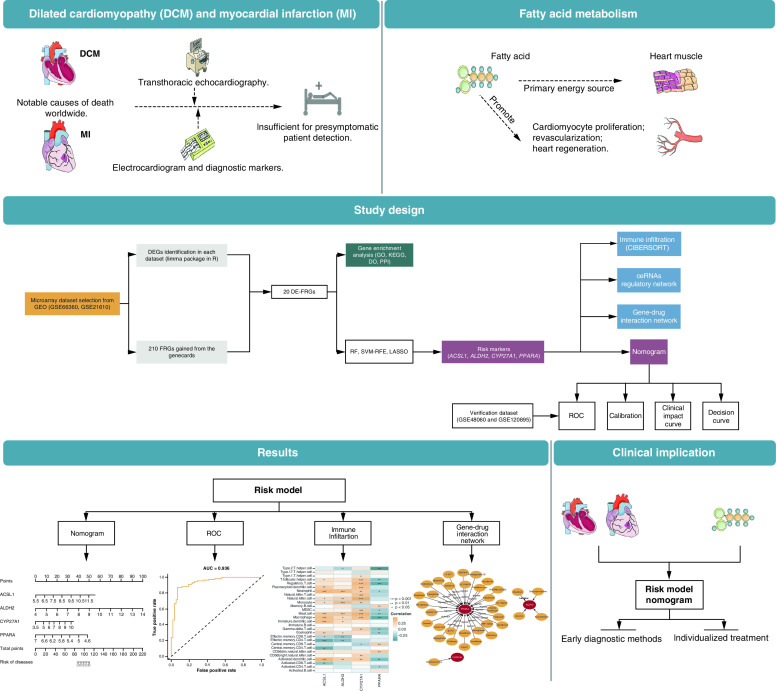

Dilated cardiomyopathy (DCM) and myocardial infarction (MI) are highly fatal cardiovascular diseases that pose significant public health threats globally [1,2]. DCM is characterized by heart muscle dilation and dysfunction, commonly ending in heart failure (HF) [1–3]; MI is caused by a sudden blockage of blood flow to the heart, leading to tissue damage and HF failure [4]. Nevertheless, current diagnostic tools that fail to predict DCM before changes in left ventricular ejection fraction [5,6] and MI before its sudden onset [4,7], are inadequate in detecting presymptomatic patients.

DCM and MI are inextricably linked. They share a few risk factors in common, including infections, inflammation and family history [8,9]. Moreover, overexpression of MI-associated transcripts has been observed in patients with inflammatory DCM [10]. Considering these observations, characterizing genetic profiles to construct a shared risk prediction model may hold promising implications for early intervention improvement.

Fatty acid, as the primary energy source for heart muscle [11], may substantially contribute to the pathogenesis of DCM and MI. Decreased expression of PPARα (fatty acid metabolism related protein) caused by doxorubicin may induce DCM [12]. Administration of eicosatetraenoic acid improved cardiac metabolism, attenuated histological remodeling and resulted in functional improvements after MI [13]. Identifying novel predictive genes in this pathway may provide valuable insights into disease pathogenesis and innovative therapeutic strategies development.

The present study aims at constructing a shared risk prediction model for DCM and MI related to fatty acid metabolism. The association between risk signatures and infiltrating immune cells, drugs and competing endogenous RNA (ceRNA) was also analyzed, which may assist in developing personalized preventive strategies to reduce the burden of cardiovascular disease.

## Materials & methods

### Data collection & data processing

First, GSE21610, GSE66360, GSE120895 and GSE48060 were downloaded as candidate datasets from the Gene Expression Omnibus (GEO) database (www.ncbi.nlm.nih.gov/geo/). The basic information of retrieved datasets is shown in Table 1. Additional clinical information for the dataset can be found in Supplementary Table 1. The raw data were normalized with quantile normalization and transformed into a base two logarithmic scale using the R package ‘LIMMA.’ Subsequently, a principal component analysis was performed to verify their quality [14]. Then, we combined GSE21610 and GSE66360 as the train set and GSE120895 and GSE48060 as the test set. The R package ‘sva’ [15] removed the merged dataset’s batch effects, and principal component analysis verified the quality (Supplementary Figure 1).

**Table 1.** Data sets implemented for analysis.

Study type	Accession ID	Platforms	Control samples	Disease samples	Disease type	Ref.
Array	GSE21610	GPL570 [HG-U133_Plus_2]	8	60	DCM	[16]
Array	GSE66360	GPL570 [HG-U133_Plus_2]	50	49	MI	[17]
Array	GSE120895	GPL570 [HG-U133_Plus_2]	8	47	DCM	[18]
Array	GSE48060	GPL570 [HG-U133_Plus_2]^1^	21	31	MI	[19]

MI: Myocardial infarction.

### Differential expression analysis

Differentially expressed genes (DEGs) between control (healthy samples) and disease (DCM or MI samples) group in the training set were identified by R packages ‘LIMMA’. DEGs with the p-value < 0.05 and |log2 FC| >1 were statistically significant. A total of 210 FRGs with a Relevance score >32 were obtained from the Genecards database. The intersection of obtained FRGs and DEGs yielded a set of DEGs associated with fatty acid metabolism (DE-FRG), visualized by R packages ‘LIMMA’, ‘pheatmap’ and ‘ggpubr’ [14,20,21].

### Gene enrichment analysis

Gene ontology (GO), Kyoto Encyclopedia of Genes and Genomes (KEGG) and Gene Set Enrichment Analysis (GSEA) were conducted with the use of the R package ‘clusterProfiler’ [22] to explore the function and pathway of the DE-FRGs. Disease ontology (DO) enrichment analysis was performed with the R package ‘DOSE’. p < 0.05 was considered to show the statistical significance.

### Construction & validation of the risk nomogram

Three algorithms were applied to the DE-FRGs for screening risk biomarkers for DCM and MI, including the random forests (RF) [23,24], the least absolute shrinkage and selection operator (LASSO) logistic regression [25] and the support vector machine recursive feature elimination (SVM-RFE) [26]. The RF algorithm was adopted with the R package ‘randomForest.’ The LASSO logistic regression investigation was conducted with R package ‘glmnet,’ and minimal lambda was considered optimal. Later, three screening results were intersected to obtain risk biomarkers, visualized with a nomogram. The test set was used for in-depth testing of the efficacy of key biomarkers. The receiver operating characteristic curve (ROC), calibration curve, clinical impact curve and decision curve were used to judge the predictive ability of the nomogram.

The train set and the test set were assessed based on the investigation of ROC curves, and the area under the curve (AUC) was calculated to evaluate the predictive effect achieved by the algorithms. These steps were achieved via R package ‘dplyr’ [27], ‘timeROC’ [28], ‘rms’ [29] and ‘ROCR’ [30]. Following this, the calibration curve (relationship between observation probability and prediction probability) and clinical impact curve were performed to evaluate the degree of consistency between observed and predicted outcomes. The decision curve analysis examined the theoretical relationship between patients’ threshold death or relapse probability and the relative value of false-positive and false-negative results to evaluate the nomogram benefit further [31]. These steps were achieved via R package ‘Hmisc’ [32], ‘lattice’ [33] and ‘Formula’ [34].

### Evaluation & correlation analysis of infiltration related immune cells

The CIBERSORT website filters 28 kinds of the immune cell matrix. According to p < 0.05, the immune cell infiltration matrix was obtained. The present study adopted the R package ‘corrplot’ for drawing the correlation heatmap for visualizing the correlation of 28 kinds of infiltrating immune cells. The ‘ggstatsplot’ and ‘ggplot2’ packages were adopted to analyze the Spearman relationship between characteristic risk markers and immune infiltrating cells and visualize the result.

### Construction of the gene–drug interaction network

Drugs and their target genes were downloaded from the gene-drug interaction database (DGIdb v3.0, www.dgidb.org) [35]. Cytoscape v3.9.1 was applied to extend the gene–drug interaction network (https://cytoscape.org/).

### Construction of ceRNA network

A ceRNA network was built based on three public websites, TargetScan, miRDB, and miranda, to determine whether the DE-FRGs exist in competing endogenous regulating networks mediated by long non-coding RNAs (lncRNAs) and micro RNAs (miRNAs). Visualization of the ceRNA was performed by Cytoscape v3.9.1 (https://cytoscape.org/).

### Data analysis

R v4.1.2 (R Foundation for Statistical Computing, Vienna, Austria, www.r-project.org/) and RStudio v1.1.463 (Integrated Development for R. RStudio, Inc., MA, USA, www.rstudio.com/) were operated to perform data analysis. Significance evaluation was fulfilled by *t*-test for distributed data and by Wilcoxon Mann–Whitney test for other data. The Spearman correlation coefficient was calculated to identify the associations between the DE-FRGs and 28 infiltrating immune cells. A multivariate logistic regression model was established to estimate the hazards ratio along with 95% CI. Analysis with a p-value is statistically significant when it is <0.05 and considered statistically highly significant if it is <0.01.

## Results

### Research flow & the collection of DE-FRGs

**Figure 1.**
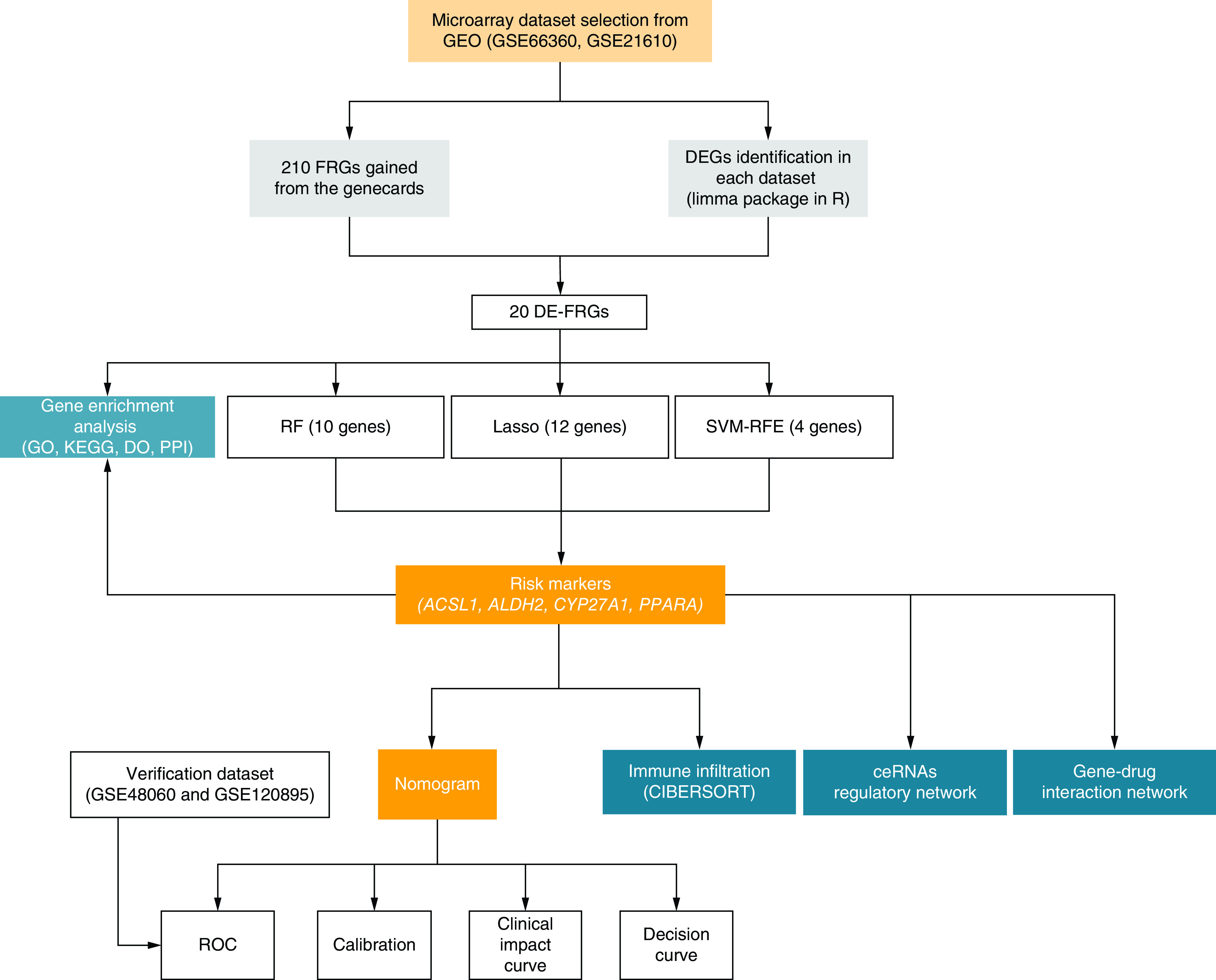
The analysis process. GO: Gene ontology; KEGG: Kyoto Encyclopedia of Genes and Genomes; GSEA: Gene Set Enrichment Analysis; PPI: Protein–protein Interaction.

**Figure 2.**
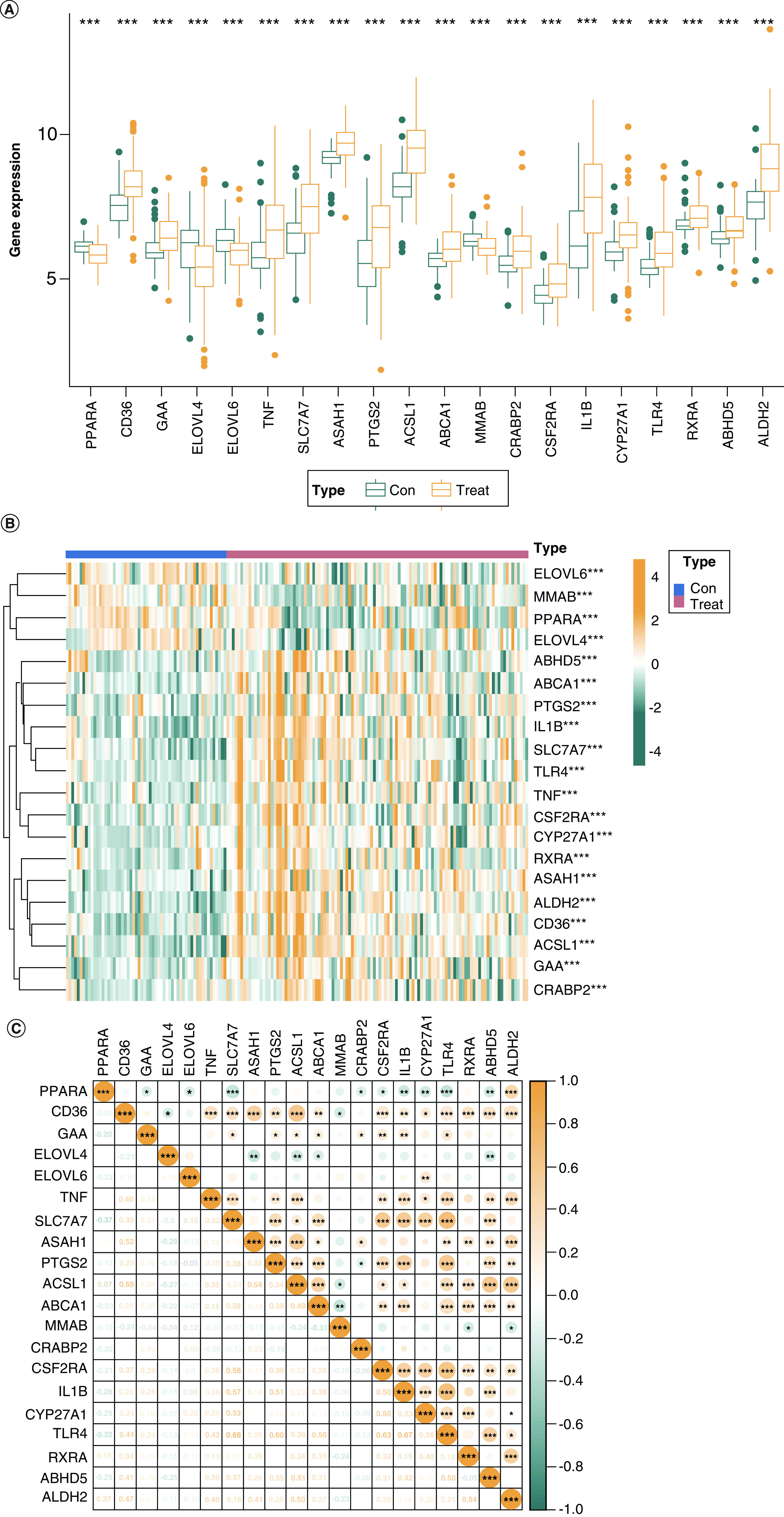
Expression of differentially expressed fatty acid metabolism related genes in dilated cardiomyopathy and myocardial infarction. **(A)** Box plots showing the differential gene expression in diseases and control samples. **(B)** Heat map showing the enrichment of 20-DE-FRG in control samples and disease samples. **(C)** Scatter plot showing the correlation among 20-DE-FRG. *p < 0.05; **p < 0.01; ***p < 0.001, determined by Kruskal–Wallis test. DE-FRG: Differentially expressed fatty acid metabolism related gene.

After downloading the dataset from GEO, quality control and normalization of the gene expression matrix were performed. Subsequently, differential analysis was executed for DEGs, intersecting with FRGs to generate 20 DE-FRGs. Among them, four were identified as risk biomarkers by the SVM-RFE algorithm, 12 by the LASSO algorithm, and 10 by the RF algorithm, respectively. After the intersection of these genes, four DE-FRGs were finally selected for nomogram. We then conducted KEGG, GO and DO analysis and established a protein–protein Interaction (PPI) network of the 20 DE-FRGs. Immuno-infiltration analysis, ceRNA regulatory network construction and gene–drug interaction network construction were undertaken based on the 4-DE-FRG (Figure 1). Differential gene expression analysis was performed on the control and disease groups of the train set, and a total of 20 DE-FRGs were obtained. In the disease groups, the *PPARA, CD36, ELOVL4, PTGS2, ACSL1, MMAB, CSF2RA, CYP27A1, RXRA, ABHD5* and *ALDH2* were downregulated and *GAA, ELOVL6, TNF, SLC7A7, ASAH1, ABCA1, CRABP2, IL1B* and *TLR4* were upregulated (Figure 2A & B).

### Construction & validation of the risk nomogram based on DE-FRGs

**Figure 3.**
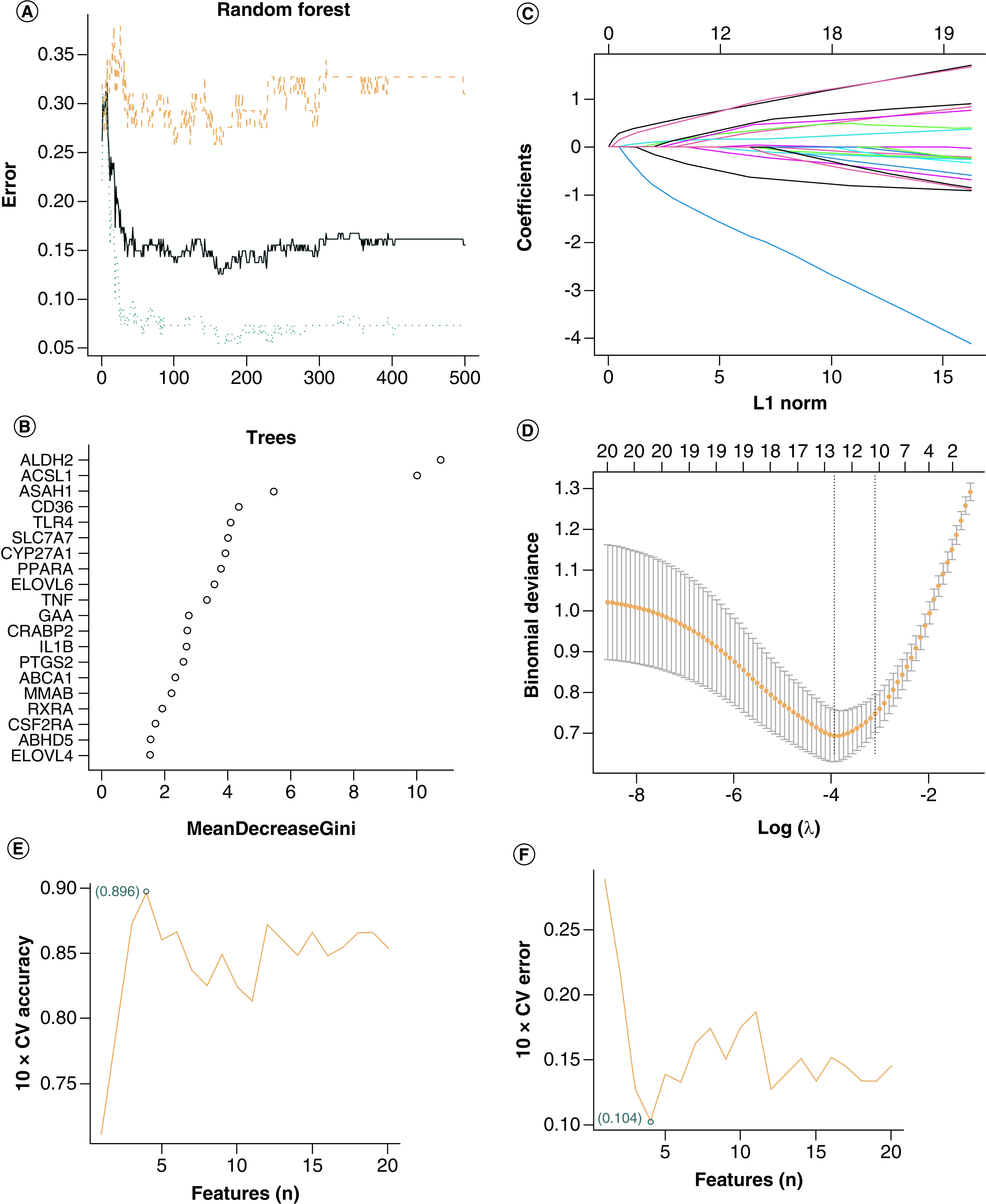
Screening of risk markers via comprehensive strategies. **(A)** Based on RF algorithm to screen biomarkers. **(B)** The mean decrease Gini of risk factors associated DE-FRGs based on RF analysis. **(C & D)** LASSO logistic regression algorithm to screen risk markers. **(E & F)** Based on SVM-RFE to screen biomarkers. DE-FRG: Differentially expressed fatty acid metabolism related gene; SVM-RFE: Support vector machine recursive feature elimination.

Among the 20 DE-FRGs, ten key biomarkers were identified by the RF algorithm with an importance score greater than 3 (Figure 3A & B). Twelve genes were identified as important biomarkers with LASSO logistic regression algorithm (Figure 3C & D). Later, four genes were identified as key biomarkers by the SVM-RFE algorithm (Figure 3E & F). Following the intersection, four genes (*ACSL1, ALDH2, CYP27A1* and *PPARA*) were obtained for subsequent analysis.

**Figure 4.**
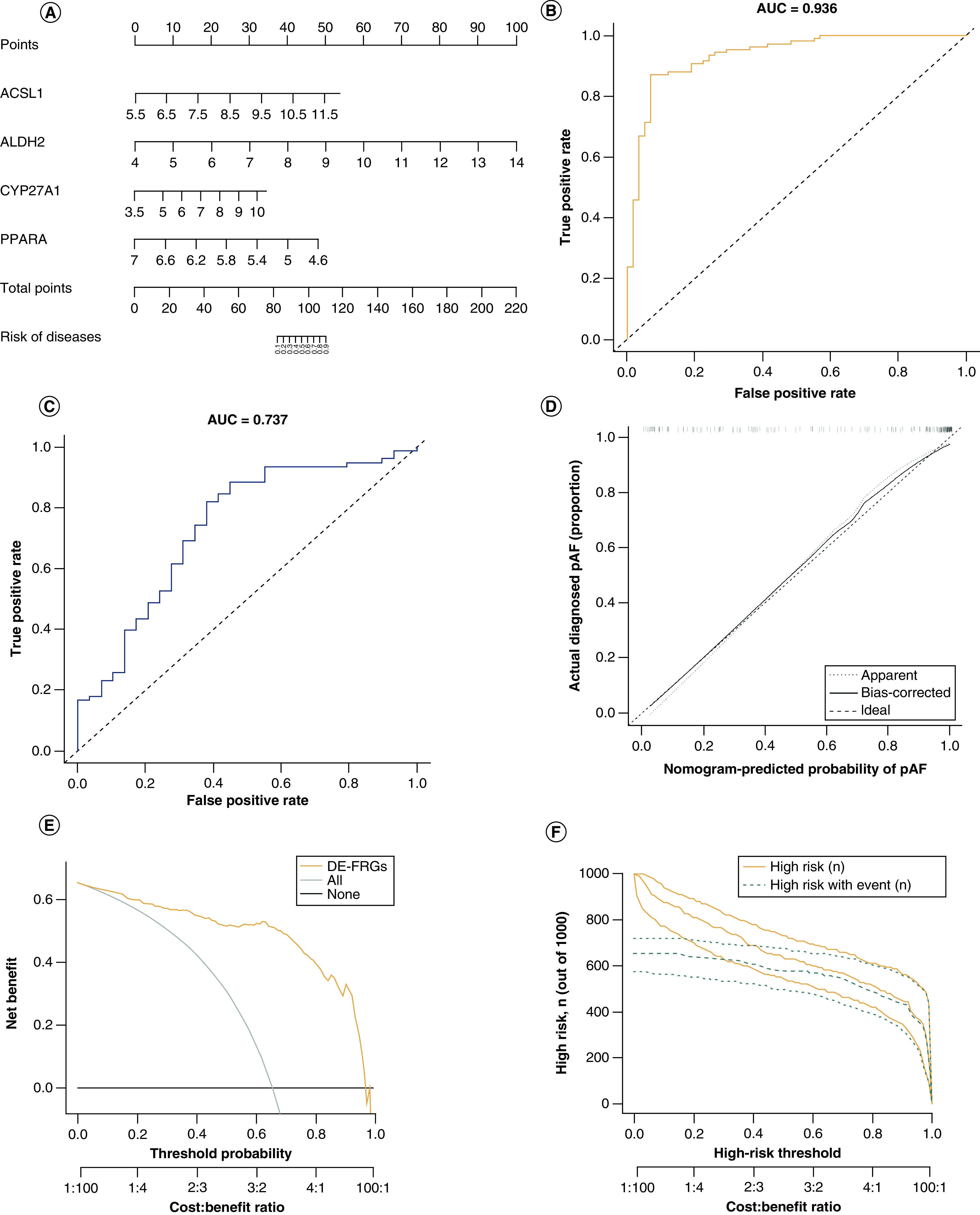
Nomogram, receiver operating characteristic curves, clinical impact curve and decision curve for 4-differentially expressed fatty acid metabolism related gene risk model. **(A)** Nomogram for the 4-DE-FRG risk model. **(B)** ROC curves of the train set. **(C)** ROC curves of the test set. **(D)** Calibration. The disease probabilities predicted by the nomogram are plotted on the x-axis, and the actual disease probabilities are plotted on the y-axis. **(E)** Decision curve. The abscissa of this graph is the threshold probability, and the ordinate is the net benefit. **(F)** Clinical impact curve. The green curve indicates the number of people classified as positive (diseases) by the model at each threshold probability; the orange curve is the number of true positives at each threshold probability. AUC: Area under the curve; DE-FRG: Differentially expressed fatty acid metabolism related gene; ROC: Receiver operating characteristic.

A risk model based on the 4-DE-FRG (*ACSL1, ALDH2, CYP27A1* and *PPARA*) was constructed, and a nomogram (Figure 4A) was drawn for clinical ease of use. According to the measurements of the 4 DE-FRGs expression levels in the patients blood, the user can find them on the corresponding scale in the nomogram and project them onto the top scale to read the points for each variant. The sum of each point is the total number of points, and the risk of disease for this patient can be obtained by projecting the total points downwards. The AUC of the train set was 0.936 (Figure 4B), indicating that the risk nomogram had an excellent accuracy of predictive value. It was also confirmed by the test set (AUC = 0.737) (Figure 4C). The calibration curves of the nomogram showed a good agreement in the train set (Figure 4D). In addition, the clinical impact curves and the decision curve were proposed to assess the clinical usefulness of the risk prediction nomogram (Figure 4E & F).

### Enrichment analysis of DE-FRGs in DCM & MI

**Figure 5.**
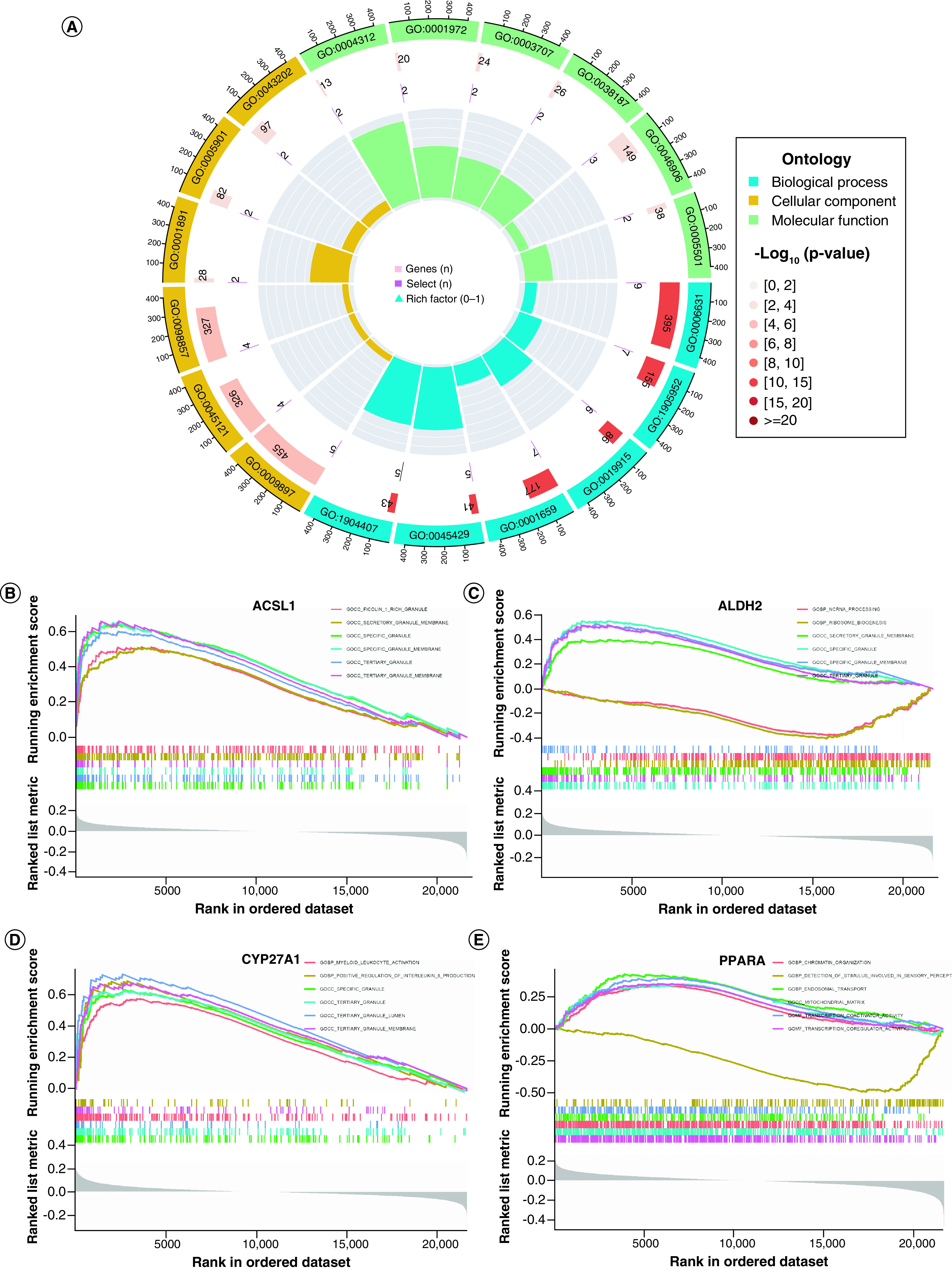
The results of gene ontology analyses. **(A)** GO analyses results of 20 DE-FRGs. **(B–E)** Sequentially, the significantly enriched GO terms for *ACSL1*, *ALDH2*, *CYP27A1* and *PPARA*. DE-FRG: Differentially expressed fatty acid metabolism related gene; GO: Gene ontology.

GO analysis revealed that DE-FRGs were mainly enriched in biological processes related to lipid metabolisms and small-molecule transport, such as fatty acid metabolic process, external side of plasma membrane and fatty acid synthase activity (Figure 5A). The enriched GO terms for individual 4-DE-FRG were shown separately in Figure 5B–E.

Similarly, KEGG analysis showed that DE-FRGs were mainly enriched in lipid metabolism pathways, such as adipocytokine signaling pathway, PPAR signaling pathway and lipid and atherosclerosis (Figure 6A). The enriched KEGG terms for individual 4-DE-FRG were shown separately in Figure 6B–E. These enrichments in DCM or MI suggested that their inhibitors could be considered potential therapeutic strategies for patients with DCM or MI.

**Figure 6.** The results of functional enrichment analyses. **(A)** Pathway analysis results of 20 DE-FRGs; **(B–E)** The significantly enriched KEGG terms for *ACSL1, ALDH2, CYP27A1* and *PPARA*, sequentially. DE-FRG: Differentially expressed fatty acid metabolism related gene.

**Figure 7.**
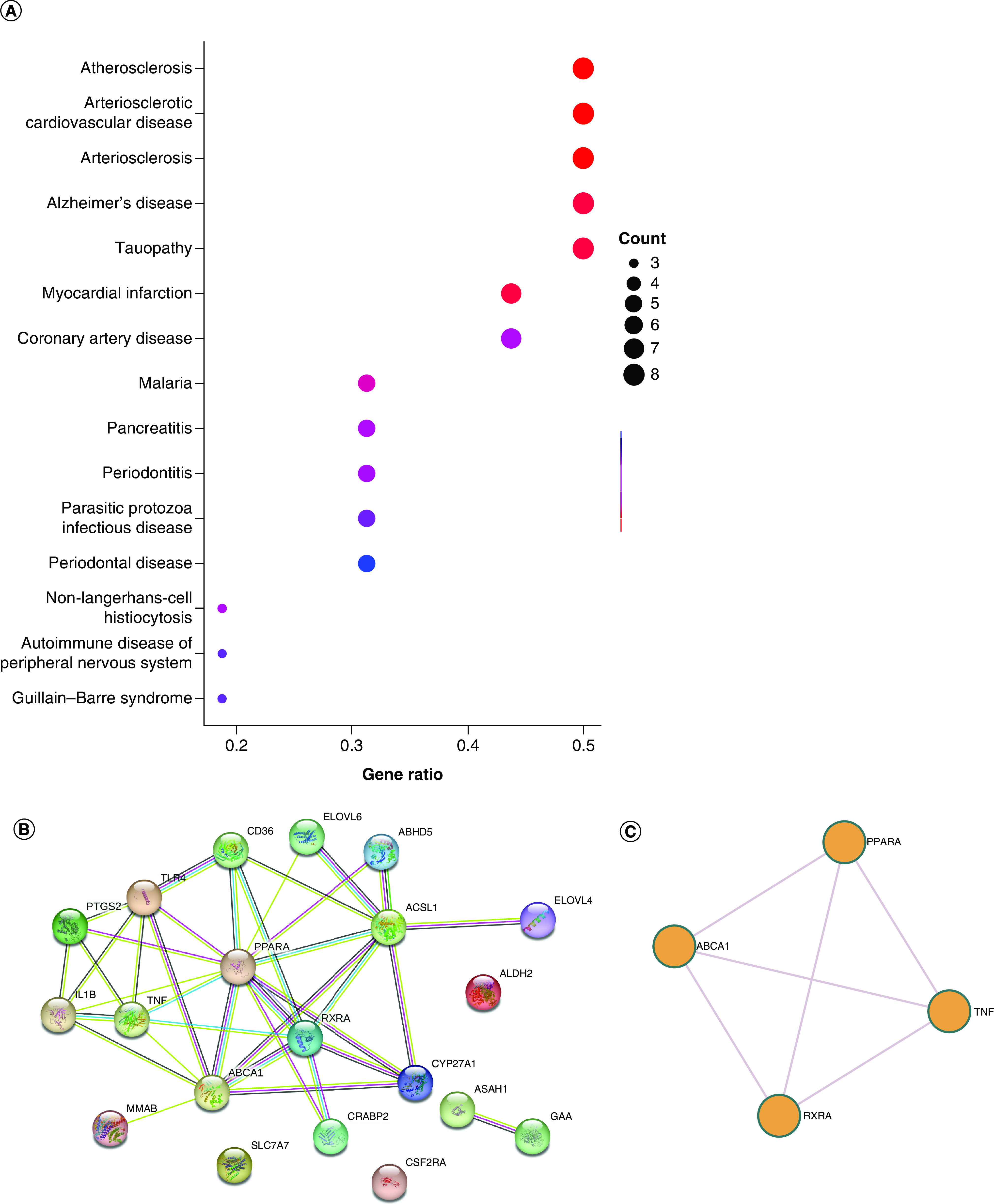
Disease ontology analysis and the PPI network of differentially expressed fatty acid metabolism related gene in dilated cardiomyopathy and myocardial infarction. **(A)** DO analysis of the 20 DE-FRGs. **(B)** The PPI network on 20 DE-FRGs. **(C)** A network of enriched terms analyzed by Metascape. DE-FRG: Differentially expressed fatty acid metabolism related gene; DO: Disease ontology. PPI network: Protein–protein Interaction network.

DO analysis presents 4 DE-FRGs associated diseases to facilitate further study of disease mechanisms. The top 15 most enriched diseases are atherosclerosis, arteriosclerotic, cardiovascular disease, arteriosclerosis, myocardial infarction, Alzheimer’s disease, tauopathy, malaria, coronary artery disease, pancreatitis, non-Langerhans-cell histiocytosis, periodontitis, parasitic protozoa infectious disease, autoimmune disease of peripheral nervous system, Guillain–Barre syndrome and periodontal disease (Figure 7A). The STRING database was used to construct the PPI interaction network of 20 DE-FRGs (Figure 7B). It was revealed that *PPARA* might be the hub node in all 20 DE-FRGs. Additionally, we performed enrichment analysis using Metascape (Figure 7C), and the result is similar to Figure 7B.

### Analysis of immune infiltration

**Figure 8.**
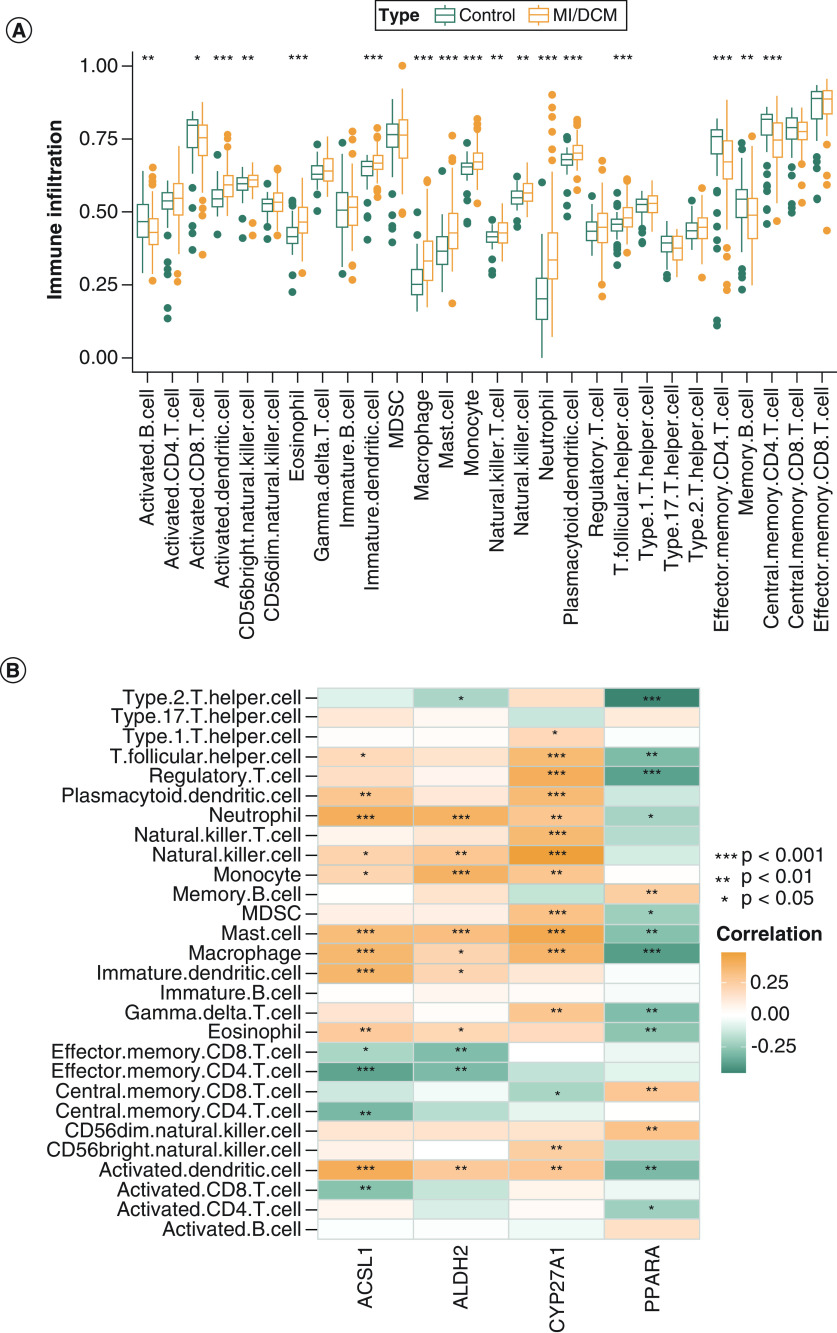
Visualization of immune cell infiltration. **(A)** Box plot of the proportion of 28 types of immune cells. **(B)** Heatmap of the correlation between risk markers and infiltrating immune cells. *p < 0.05; **p < 0.01; ***p < 0.001, determined by Kruskal–Wallis test.

Seventeen immune cells significantly differed between MI or DCM patients and healthy individuals (Figure 8A). Among them, activated B cell, activated CD8^+^ T cell, effector memory CD4^+^ T cell, memory B cell and central memory CD4^+^ T cell were lowly expressed in the disease group. *PPARA* was mainly positively correlated with adaptive immune cells and negatively correlated with innate immune cells, while *ACSL1*, *ALDH2* and *CYP27A1* were the opposite. (Figure 8B).

### Construction of regulatory gene–drug network

**Figure 9.**
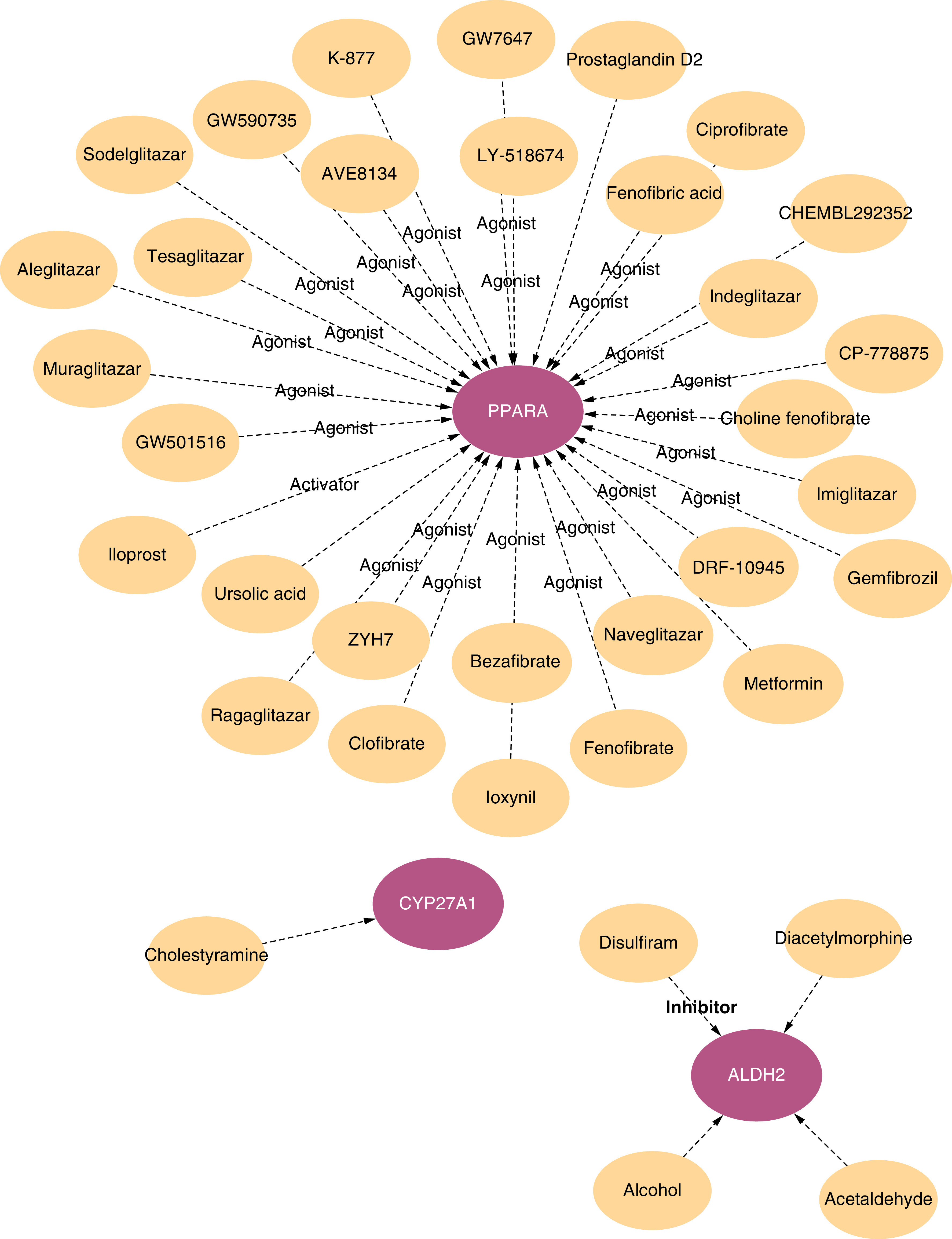
Gene–drug interaction network constructed by cytoscape. The red ovals represent the 4-DE-FRG affected by drugs. The orange ovals represent DCM/MI associated drugs. DCM: Dilated cardiomyopathy; DE-FRG: Differentially expressed fatty acid metabolism related gene; MI: Myocardial infarction.

A gene-drug network was created to provide more detailed information to illustrate the complex associations between drugs and the 4-DE-FRG targets. According to Figure 9 and Supplementary Table 2, multiple drugs have agonist roles in *PPARA*.

### Construction of ceRNA network

**Figure 10.**
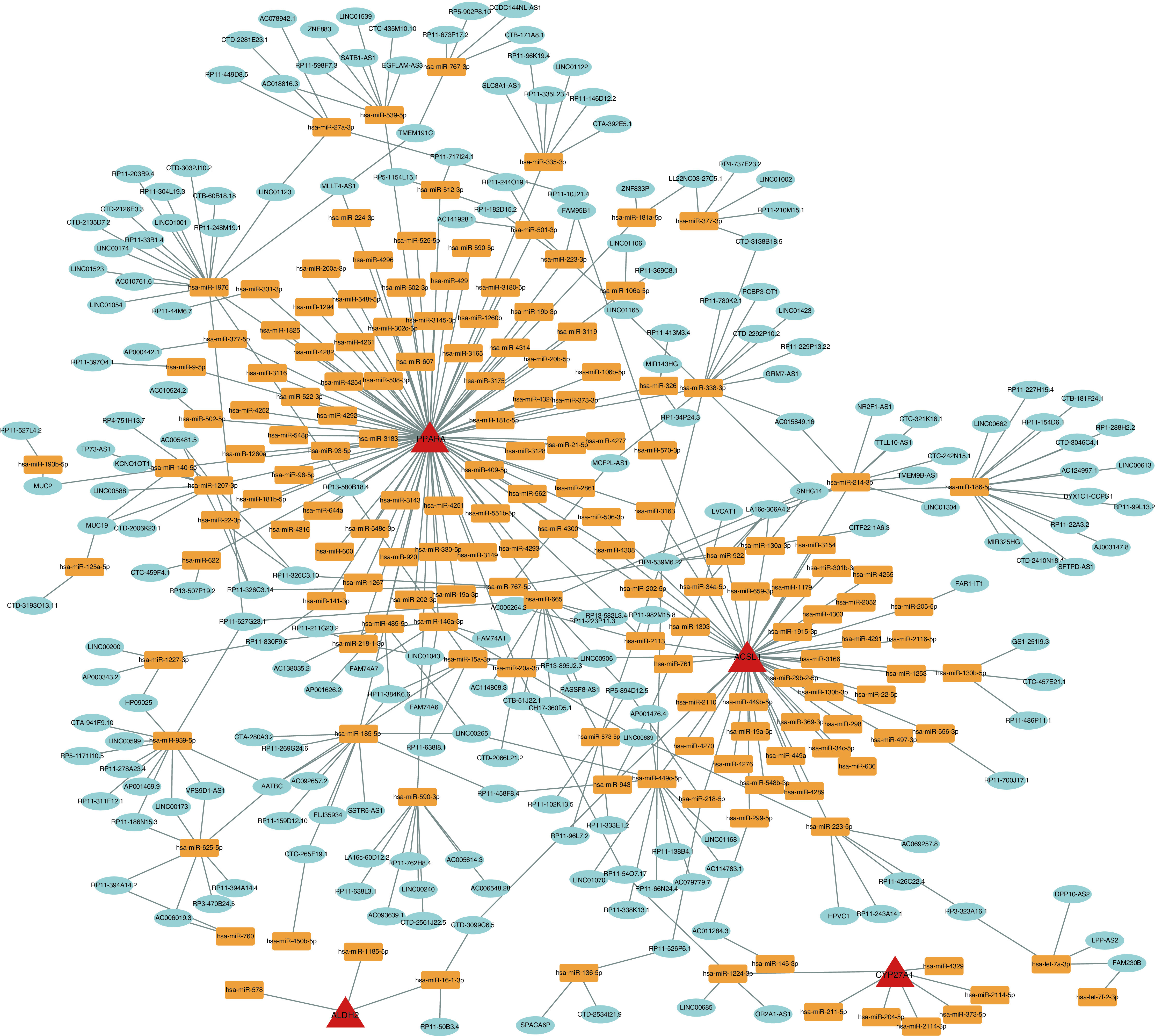
ceRNA network in dilated cardiomyopathy and myocardial infarction. The red triangles represent 4-DE-FRG. The orange squares represent miRNAs. The green ovals represent mRNAs or lncRNAs. DE-FRG: Differentially expressed fatty acid metabolism related gene.

To better explain the interactions of multiple RNA types at the gene level, three public websites, TargetScan, miRDB and miranda, were used to predict potential combinations between 4-DE-FRG and transcription factors. As shown in Figure 10, all edges contained 412 nodes, 150 positively correlated miRNA-mRNA edges and 262 positively correlated miRNA-lncRNA edges.

## Discussion

The lack of effective diagnostic methods for DCM and MI presymptomatic patients makes it important to develop a stable risk model. Our study constructed a risk prediction nomogram for DCM and MI based on the 4-DE-FRG (*ACSL1, ALDH2, CYP27A1* and *PPARA*), showing good performance in external validation (AUC = 0.737). Notably, all the four genes were upregulated in disease groups. Later pathway enrichment analysis uncovered that the 4 DE-FRGs are associated with lipid metabolism and small molecule transport pathways. In addition, the 4-DE-FRG was highly correlated with infiltrating immune and 35 drug candidates in DCM and MI.

The heart is a highly energy demanding organ with fatty acids as its leading energy supplier. Davila *et al.* found that cardiac fatty acid metabolism was decreased in DCM patients [36]. Malonic acid can promote cardiomyocyte proliferation and heart regeneration in MI mice [37]. Moreover, short-chain fatty acids are more easily oxidized and utilized in failing hearts [38], representing a promising metabolic target.

*PPARA* regulates lipid metabolism, making it the core of atherosclerotic plaque formation [39]. *PPARA* is protective against DCM and MI [40,41]. The *PPARA* agonist fenofibrate attenuates left ventricular diastolic abnormalities and systolic dysfunction to delay HF's progression and improve survival in HF animal models [40,42]. That is consistent with our belief that *PPARA* is protective. Exploring the mechanisms of *PPARA* in DCM and MI may be a breakthrough.

Seventeen immune cells significantly differed between MI or DCM patients and healthy individuals. Neutrophils may be a risk marker for the severity of HF in DCM patients [43], and their elevation is related to poor prognosis in MI [44]. Mast cells are influential in ventricular remodeling and HF progression in DCM mice [43], and their stabilizers reduce ischemia/reperfusion injury in MI [45,46]. Blockade of NK cells and their receptors reduces inflammation and injury in DCM [47–50]; similarly, NK cells increase inflammation in the infarct zone during MI. The function of adaptive immune cells during MI, such as CD4^+^ T [51,52], CD8^+^T [53,54], and Th_2_ [55,56], remains elusive. Few studies have considered how the 4 DE-FRGs affect DCM and MI inflammation, which may be necessary as the essential polyunsaturated fatty acids produce the most potent inflammatory mediators of MI [57,58]. According to our results and the research mentioned above, *ACSL1*, *ALDH2* and *CYP27A1* were positively associated with more harmful cells, whereas *PPARA* was negatively associated with them. That corresponds to our nomogram that *ACSL1*, *ALDH2* and *CYP27A1* are risk factors and *PPARA* is a protective factor. However, uncertainty about the role of immune cells in DCM and MI leaves these speculations to further investigation.

By KEGG and GO analysis, we found that lipid metabolism and small molecule transport-related pathways, such as the fatty acid metabolic process, the external side of the plasma membrane and the fatty acid synthase activity, were enriched in DCM and MI. By constructing a gene–drug regulatory network, we also revealed that thirty agonists and activators were sensitive to *PPARA*, which might be a promising drug target against DCM and MI. In contrast, the correlation between other genes and existing drugs requires more investigation. Noncoding RNAs have been evaluated as potential diagnostic and therapeutic targets for various cardiovascular diseases [59,60], including DCM [61,62]. Simultaneously, the lncRNA-miRNA-mRNA axis can link lncRNAs to various regulatory functions in MI [63]. Therefore, constructing ceRNA regulatory networks may lead to potential diagnostic and therapeutic targets. Our result shows that *PPARA* was regulated by the maximum number of miRNAs among the 4 DE-FRGs, suggesting that it might be a hub node of the diagnosis and treatment for DCM and MI.

## Limitations

First, this study is a retrospective study designed based on a public database, and the developed model lacks evidence from *in vivo* and *in vitro* experiments. More animal experiments and extensive clinical samples are needed. Second, the public datasets provided limited information on the samples, with missing data on the source of the samples, age, BMI and gender of the sample donors. Additionally, DCM is a complex disease with heterogeneous pathological mechanisms [64]. We did not consider that different etiologies expressed relevant pathways may respond differently to treatment, possibly leading to inaccurate model predictions. In the future, it is of prospective clinical value to experimentally investigate the role of immune cells and ceRNA regulatory networks in the development of DCM or MI.

## Conclusion

In this study, we constructed a 4-DE-FRG nomogram and identified potential biological targets for the early diagnosis and treatment of DCM and MI.

**Summary points**

- A risk model based on the 4-differentially expressed fatty acid metabolism related gene (DE-FRG) (*ACSL1, ALDH2, CYP27A1* and *PPARA*) was constructed. The area under the curve of the train set was 0.936 (Figure 4B), and that of the test set was 0.737 (Figure 4C).
- *PPARA* was mainly positively correlated with adaptive immune cells and negatively correlated with innate immune cells, while *ACSL1*, *ALDH2* and *CYP27A1* were the opposite. That corresponds to our nomogram that *ACSL1, ALDH2* and *CYP27A1* are risk factors and *PPARA* is a protective factor.
- Lipid metabolism and small molecule transport-related pathways, such as the fatty acid metabolic process, the external side of the plasma membrane and the fatty acid synthase activity, were enriched in dilated cardiomyopathy and myocardial infarction.
- The PPI interaction network of 20 DE-FRGs revealed that *PPARA* might be the hub node in all 20 DE-FRGs. Multiple drugs have agonist roles in *PPARA*. *PPARA* was regulated by the maximum number of miRNAs among the 4 DE-FRGs.
