# Supplementary figures and images for: Identification of shared fatty acid metabolism related signatures in dilated cardiomyopathy and myocardial infarction
Source: Future Sci OA. 2023 Mar 28;9(3):FSO847. doi: 10.2144/fsoa-2023-0008 (PMC10088053; doi:10.2144/fsoa-2023-0008)

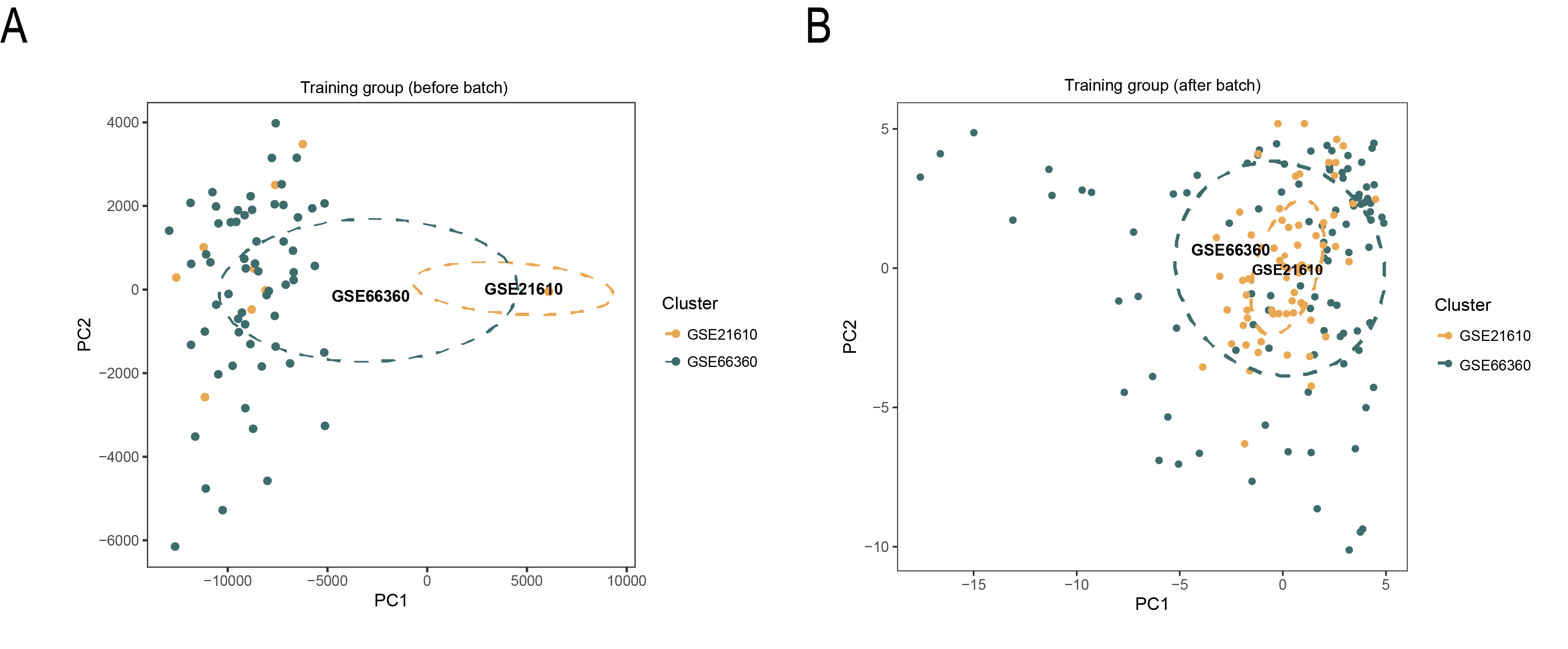

Supplement: Supplementary file 1 [file fsoa-09-847-s1.png]
